# Effectiveness of self-efficacy-enhancing interventions on rehabilitation following total hip replacement: a randomized controlled trial with six-month follow-up

**DOI:** 10.1186/s13018-022-03116-2

**Published:** 2022-04-10

**Authors:** Ya Meng, Bo Deng, Xiaoyu Liang, Jiangzhen Li, Liuyi Li, Jinxia Ou, Shuping Yu, Xingxian Tan, Yumei Chen, Meifen Zhang

**Affiliations:** 1 School of Nursing, Sun Yat-Sen University, 74 Zhongshan Road 2, Guangzhou, 510080 Guangdong China; 2 Nursing Department, Sun Yat-Sen University Cancer Center, Guangzhou, Guangdong China; 3 Department of Orthopedics, Foshan Hospital of Traditional Chinese Medicine, Foshan, Guangdong China; 4 CRM China of Medtronic, Shanghai, China; 5 Nursing Department, Foshan Hospital of Traditional Chinese Medicine, 6 Qinren Road, Foshan, Guangdong China

**Keywords:** Hip replacement, Self-efficacy, Compliance, Quality of life, Rehabilitation

## Abstract

**Background:**

As the world’s population ages, hip replacement, a routine treatment for arthritis, has become more common. However, after surgery, rehabilitation has some limited effectiveness with postoperative complications and persistent impairments. This study aimed to explore the effect of a self-efficacy-enhancing intervention program following hip replacement on patients’ rehabilitation outcomes (self-efficacy, functional exercise compliance, hip function, activity and social participation, anxiety and depression, and quality of life).

**Methods:**

A prospective randomized controlled trial with a repeated-measures, two-group design was conducted in a grade A general hospital in Guangdong Province, China. A total of 150 participants with a unilateral total hip replacement were recruited via convenience sampling. Participants were randomly assigned to either the self-efficacy enhancing intervention group (*n* = 76) or the control group (*n* = 74). The intervention encompassed a face-to-face education before discharge and four telephone-based follow-ups in six months after surgery. Researchers collected baseline data on one to three days after surgery, and outcomes data were collected one, three, and six months after surgery.

**Results:**

Average age (deviation) in intervention and control group were 58 (10.32) and 59 (10.82), respectively. After six months, intervention group scored 86.83 ± 5.89 in rehabilitation self-efficacy, significantly higher than control group (72.16 ± 6.52, *t* = -10.820, *p* < 0.001) and their hip function has turned to “excellent” (90.52 ± 4.03), while that of the latter was limited to a “middle” level (78.47 ± 7.57). Statistically significant differences were found in secondary outcomes (*p* < 0.001). The advantage of intervention in improving quality of life was seen in the long term rather than in the early postoperative period.

**Conclusions:**

The self-efficacy-enhancing intervention performed by nurses induced better exercise compliance and physical, psychological, and social functions after hip replacement compared with routine care. We recommend such interventions to be combined with routine care soon after hip replacement. Further research should focus on the social participation of patients with hip replacement.

*Trial registration* Retrospectively registered at Chinese Clinical Trial Registry (31/01/2020, No. ChiCTR2000029422, http://www.chictr.org.cn/index.aspx).

**Supplementary Information:**

The online version contains supplementary material available at 10.1186/s13018-022-03116-2.

## Background

Hip replacement or hip arthroplasty, a surgical procedure using a prosthesis to entirely or partially replace a damaged hip joint, has been routinely used to relieve hip pain and joint stiffness over the past 60 years [1]. More than one million total hip replacements (THR) are annually performed worldwide [2]. In 2018, over 550,000 hip replacements were performed in the USA and 150 000 were done in France [3]. In 2019, approximately 600 000 hip replacements were performed in China, with the rate increasing 20% per year [4].

Although hip replacement can relieve pain and restore joint function, rehabilitation has limited effectiveness with postoperative complications and persistent impairments. The reported incidence of prosthesis dislocation and deep vein thrombosis after THR could reach up to 10% and 12.8%, respectively [5, 6], leading to 5.5% of 30-day readmission rate [7]. Postoperative activity levels are disappointingly low in many patients. Around 20% of patients are socially isolated following surgery, and 7%-23% of patients reported an unfavorable long-term pain [8].

Previous studies showed postoperative systematic exercise promoted rehabilitation effectively, reducing adverse complications such as deep vein thrombosis and prosthesis dislocation, strengthening muscle, as well as improving range of joint motion and walking speed [9]. These findings demonstrate that exercise compliance is related to their rehabilitation outcomes, with better compliance leading to fewer complications and better joint function.

With the increasing tendency to perform Enhanced Recovery After Surgery (ERAS), for hip replacement, length of stay has reduced to a few days [10]. The downside is that patients have to accomplish the functional exercise at home, which lasts for 3 to 6 months. During the lengthy home-based rehabilitation, patients’ compliance is often not ideal because of limited support. Compliance with the required exercise was less than 50% in hospital, while one month after discharge, fewer than 30% of those who had undergone hip replacement took the initiative to continue with their exercises [11], and the ratio decreased over time [12]. The poor compliance limited the benefits of functional exercise, with a large diversity in rehabilitation [13]. Therefore, there is an urgent need for nursing to improve compliance and rehabilitation.

According to Bandura’s theory, self-efficacy, describing the degree and persistence of efforts when facing obstructions or frustrations, regulated human motivation, behavior, and well-being [14]. In the present study, self-efficacy of rehabilitation refers to the belief to accomplish specific activities needed after surgery. It has been proved to be a crucial predictor of exercise compliance, pain, and hip function after THR [15], with higher self-efficacy indicating greater knowledge, better exercise compliance, and quality of life [16]. The self-efficacy-enhancing interventions have commonly been used in some chronic disease, including cancer, coronary heart disease, diabetes, and hypertension to improve patients’ self-administration, health-related behavior, and long-term outcomes [17].

Unfortunately, although lack of positive belief of physical exercise hindered motivation and effect of exercise, there are no large-scale trials of interventions aimed at improving self-efficacy after THR. Therefore, in this study, a six-month self-efficacy-enhancing intervention was conducted to examine the effects of the program on exercise compliance, hip function, activity and participation, negative emotions, and quality of life.

## Methods

### Study design

This is a parallel group, randomized controlled trial comparing provision of self-efficacy-enhancing intervention against no provision. The control group received routine orthopedic care, including perioperative education, postoperative health manuals, functional exercise guidance, care following complications, psychological care, and regular outpatient visits and follow-up about the hip function recovery. The intervention group received a six-month self-efficacy enhancing intervention in addition to their routine care.

### Participants and data collection

Participants were recruited via convenience sampling in a grade A general hospital in Guangdong Province, China. Inclusion criteria were as follows: (1) unilateral THR for the first time; (2) at least primary education; (3) 18–75 years of age; (4) voluntary participation and close cooperation with the care plan; and (5) agreement to continue the intervention and the six-month follow-up after discharge. Individuals with severe physiological or psychological diseases or those undergoing revision joint replacement surgery were excluded.

The sample size was calculated using the formula: *N* = [1 + (*K* − 1) *ρ*] *σ*^2^( *Z*_1−*α*/2_ + *Z*_1−*β*_)^2^ / *Kδ*^2^ [18]. According to Mazoochian et al. [19], *σ* = 7.5, *δ* = 15, *ρ* = 0.8, a sample size of 55 in each group was required to detect any significant differences. The estimated sample size was 140, to allow for a 20% dropout rate in this longitudinal study. Finally, 150 participants were recruited for the study.

Patients with hip replacement were approached for recruitment by research assistants 3 days postoperatively. If patients met the inclusion criteria, agreed to participate in the study and signed informed consent, they were asked to fill out the baseline questionnaires. After baseline data collection, an independent researcher randomly assigned participants into the intervention and control groups using a randomization code generated by SPSS 20.0 software. Grouping schemes were placed into sealed and opaque envelopes to conceal randomization. Participants were not informed of which group they were in. Researchers conducting the baseline evaluation also collected subsequent assessment data, and not aware of which group patient was in.

A total of four evaluations were performed from December 2017 to December 2018, including the initial one right after recruitment and three telephone-based follow-ups in one, three, and six months after surgery. The baseline data included sociodemographic and clinical data, self-efficacy of rehabilitation, hip function, activity and social participation, anxiety and depression, and quality of life. The following assessments included the above rehabilitation outcomes and functional exercise compliance. If patients could not answer the survey independently, data collectors would read the items without providing hints and record their responses. The completed questionnaire was checked immediately for omissions and errors corrected immediately.

### Intervention

Bandura’s self-efficacy theory guided the interventions. The four strategies, individual past experience, vicarious experiences, verbal persuasion, and psychological monitoring, recommended by Bandura were incorporated into each component of the intervention. The self-efficacy enhancing intervention (SEEI) included five sessions: one hour of face-to-face education before discharge, and four telephone calls follow-up in one, two, three, and six months after surgery.

The initial face-to-face intervention was conducted in the ward of hospital following THR surgery. This session focused on building individual self-efficacy. First, nurses assessed participants’ functional exercise status and psychological condition, including their pain level, the occurrence of complications, and their knowledge of the recommended functional exercises. Second, nurses educated participants in rehabilitation exercises and the matters needing attention in their daily life. A rehabilitation handbook and video recordings of the recommended exercises were used to supplement the face-to-face education, and participants were encouraged to review these at home. Nurses and patients set the goal, functional exercise plan together, and family caregivers were also mobilized to provide encouragement and support.

The following four telephone-based health-coaching sessions took about 20 to 30 min. These sessions were designed to consolidate and enhance self-efficacy. Participants in each follow-up group were required to report the number of home exercises they had completed in the previous week, their level of pain, and their mental state. Nurses then encouraged and reinforced participant’s efforts and successes and empowered them through their support. Participants with low compliance (those who reported less than three exercise sessions per week) were encouraged to discuss the barriers that prevented them from completing the exercises as required. Tailored suggestions were given to help overcome these barriers. Nurses also provided examples of positive rehabilitation for motivation. During these sessions, rehabilitation exercise education was reinforced based on the manual and video.

Four senior orthopedic nurses qualified to bachelor’s degree level or above, with at least 10 years of orthopedic nursing experience, were selected to implement the intervention in the target hospital. Two research assistants, familiar with rehabilitation care following THR, were assigned to collect the baseline and outcome data and implement interventions. Before the study, the nurses and assistants were given training, including communication skills, the importance of consistency in the applying for the intervention program, strategies for managing potential physical and psychological problems. They also received training in the study protocol, collecting informed consent, and data assessment. The principal investigator monitored the application of the interventions through observation sampling sessions. Table 1 outlines examples of the four strategies.

**Table 1:** Outline of self-efficacy-enhancing intervention components, strategies, and specific techniques

Components	Strategies	Specific techniques
Individual past experience	Providing knowledge of functional exercise Setting achievable goals Providing positive feedback	Educating participants in rehabilitation exercise, complications, and disease Encouraging participants to observe and record their exercise behavior Conferring with participants to develop functional exercise goals at different stages, making plans on when, where, and how to engage in regular physical activities Identifying challenges of postoperative rehabilitation through discussion Providing positive feedback on accomplishments
Vicarious experience	Sharing cases of successful rehabilitation	Sharing previous success stories to build confidence Introducing the successful experiences of others to motivate participants to adhere to their rehabilitation program in the following months
Verbal persuasion	Persuasion Giving verbal encouragement and compliment	Describing the benefits of physical activities Asserting that participants have the ability to self-manage Commending participants upon their efforts and giving verbal encouragement Reinforcing participants’ past and present successes or accomplishments
Psychological monitoring	Avoiding negative emotional stimulation Helping participants to seek social support	Assessing participants’ expression of anxiety and depression Identifying individual barriers to, and resources for, physical activity Providing strategies for dealing with barriers and coping in the future (post-surgery; the significance of social support)

### Data analysis

In this study, data of patients lost to follow-up were filled by mean imputation method. Intention-to-treat was applied to determine "full analysis set," and then the full analysis set was analyzed.

SPSS 23.0 was used. An alpha level of 0.05 was applied to all statistical tests. Means and standard deviations were used to describe qualitative data, and frequencies and constituent ratios were used to describe quantitative data. The social demographic data from the intervention and control groups were tested to ensure the groups were comparable. Normality tests were performed for the scores of six outcomes. Normally distributed data were expressed as mean ± standard deviation, while skewed distributions were expressed as medians. The comparison of two groups was analyzed using repeated measurement analysis of variance and independent sample t-tests [20].

### Measures

#### Sociodemographic and clinical data

Sociodemographic data included patients’ age, gender, marital status, work status, educational level, economic income, type of medical insurance; clinical data included diagnosis, operative site, prosthetic material, and preoperative and postoperative complications.

#### Outcomes

Self-efficacy relating to functional exercise was assessed by the 12-item self-efficacy for rehabilitation scale developed by Waldrop et al. [21]. Each item was scored on an 11-point Likert scale, ranging from zero (I cannot do) to 10 (certain I can do) to describe the participant’s confidence to perform the behaviors recommended for rehabilitation following hip surgery.

The exercise compliance questionnaire has three dimensions: physical exercise compliance, exercise monitoring compliance, and initiative in seeking advice compliance [12]. Each item is rated on a four-point scale, ranging from 1 (never do) to 4 (always do). The scores were combined, with higher total scores indicating higher compliance.

The hip function was assessed using the Harris Hip Score (HHS) for hip replacement [22], a multidimensional, disease-specific, observational assessment of function. The total score is 100 points: 90–100 is excellent, 80–89 is good, 70–79 is medium, below 70 is poor. The maximum possible scores for its parts are as follows: pain (44 points), walking function (33 points), activities of daily living (14 points), and range of motion and deformity (nine points).

Activity and participation was assessed by the World Health Organization Disability Assessment Schedule II (WHO-DAS II) [23]. It contains 36 items describing limitations in six domains: cognition, mobility, self-care, getting along with others, activities of daily living, and social participation. Each item is scored at five points, ranging from 1 (no difficulty) to 5 (extreme difficulty), with higher scores indicating higher limitations in daily life.

Anxiety and depression was measured using the Chinese version of the Hospital Anxiety and Depression Scale (C-HADS) [24]. This instrument consists of 14 items and two subscales, seven for anxiety and seven for depression. Subscale scores range from zero to 21, with higher scores indicating greater distress.

Quality of life was evaluated by the 12-item Short-Form Health Survey [25], comprising 12 items for assessing physical and mental health. Lower scores indicate poorer physical or mental health.

## Results

**Fig. 1:**
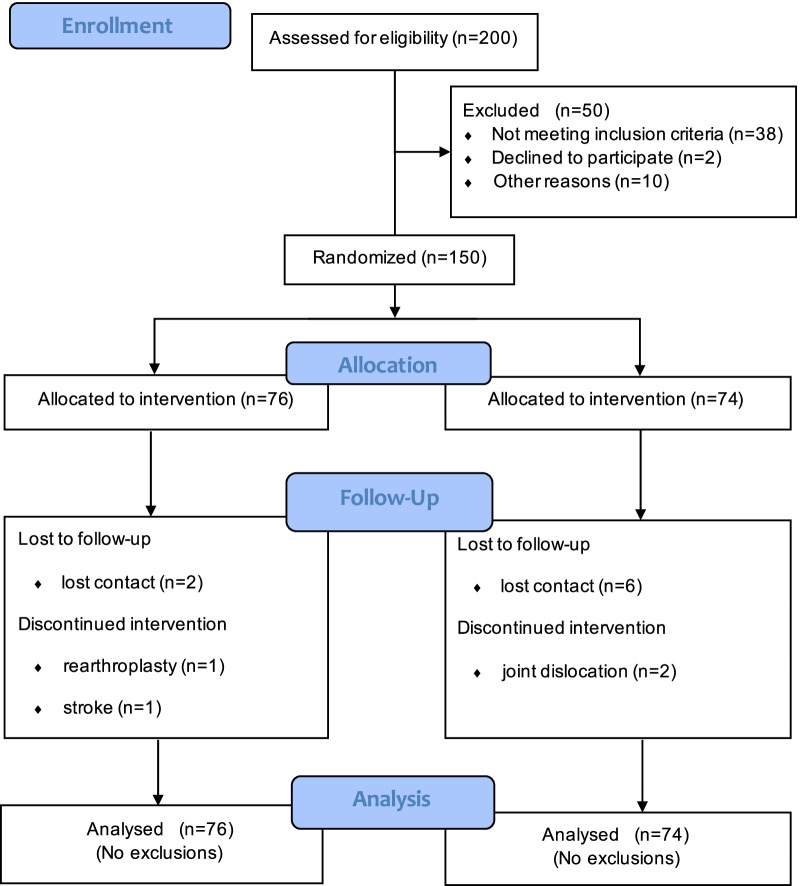
Flowchart of the randomized controlled trial

Of the 150 participants who met the inclusive criteria, 138 (72 in the intervention group and 66 in the control group) finished the six-month follow-up and all participants entered the final analysis. Figure 1 shows recruitment and withdrawal of participants. The main sociodemographic and clinical characteristics are shown in Table 2 (See other baseline data in Additional file 1: Table S1). There was no notable difference between intervention and control groups in the baseline data.

**Table 2:** Characteristics of participants (*n* = 150)

Variable	Intervention group (*n* = 76) *n* (%)/*X* ± *S*	Control group (*n* = 74) *n* (%)/*X* ± *S*	*χ*^2^*/t*	*P*
Gender			1.682	0.247
Male	28 (38.8)	35 (47.3)		
Female	48 (63.2)	39 (52.7)		
Age	58 ± 10.32	59 ± 10.82	0.626	0.532
Marital status			1.045	0.833
Unmarried	2 (2.6)	1 (1.4)		
Married	68 (89.5)	65 (87.7)		
Divorced/Widowed	6 (7.9)	8 (10.9)		
Educational level			8.347	0.132
Primary school	30 (39.5)	29 (39.2)		
Junior high school	27 (35.5)	26 (35.1)		
High school or technical secondary school	12 (15.8)	11 (14.9)		
College or bachelor’s degree	7 (9.2)	8 (10.8)		
Diagnosis			3.627	0.459
Osteoarthritis	17 (22.4)	16 (21.6)		
Osteonecrosis of the Femoral Head	20 (26.4)	18 (24.3)		
Fracture	22 (28.9)	25 (33.8)		
Congenital malformation	14 (18.4)	11 (14.9)		
Other	3 (3.9)	4 (5.4)		
Postoperative complications			5.112	0.080
No	60 (78.9)	59 (79.7)		
Yes	16 (21.1)	15 (20.3)		

**Fig. 2:**
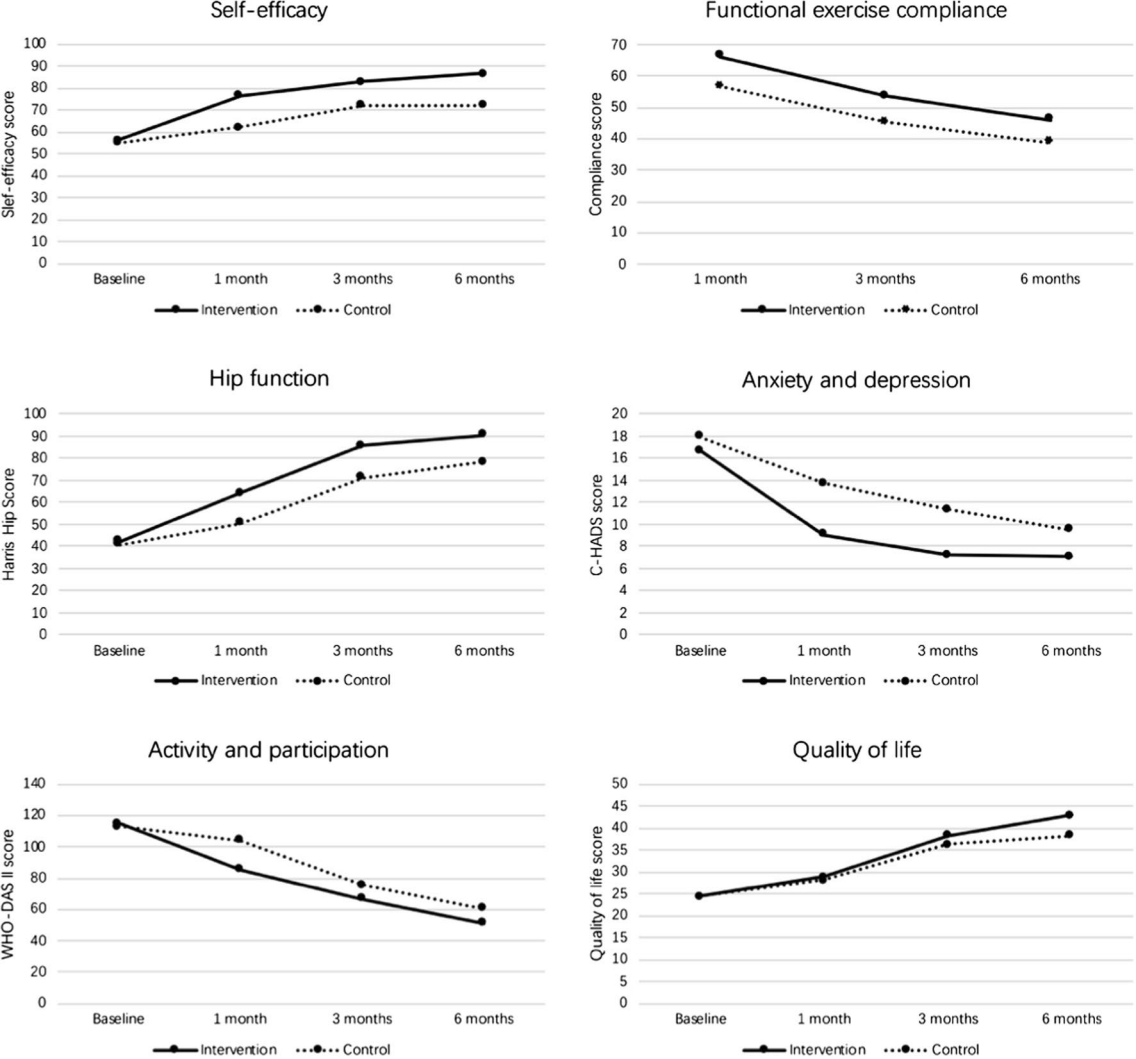
Mean scores of the outcome measures from baseline to 6 months follow-up between the intervention group (solid line) and control group (dashed line)

Figure 2 presents the tendency of the study variables. Both groups showed improved physical and psychological status, while their functional exercise compliance declined over time. Table 3 presents the results of repeated measurement analysis of variance for comparing two groups.

**Table 3:** Comparison of rehabilitation outcomes between the intervention and control groups on three tracking points (*n* = 150)

Variable	Time	Intervention group	Control group	The independent sample *t*-test		The repeated measurement analysis of variance		
				*t*	*P*		*F*	*P*
Self-efficacy of rehabilitation	T_1_	76.80 ± 4.40	62.22 ± 5.55	− 13.288	< 0.001*	^a^Group × Time	33.473	< 0.001*
	T_3_	83.08 ± 7.22	72.17 ± 7.22	− 8.140	< 0.001*			
	T_6_	86.83 ± 5.89	72.16 ± 6.52	− 10.820	< 0.001*			
Hip function	T_1_	64.26 ± 7.73	50.67 ± 6.20	− 8.981	< 0.001*	^a^Group × Time	36.790	< 0.001*
	T_3_	85.77 ± 5.61	71.11 ± 7.46	− 3.112	0.010*			
	T_6_	90.52 ± 4.03	78.47 ± 7.57	− 8.998	< 0.001*			
Activity and participation	T_1_	85.57 ± 6.94	104.17 ± 9.28	10.353	< 0.001*	^a^Group × Time	27.800	< 0.001*
	T_3_	66.86 ± 9.95	75.74 ± 8.63	4.406	< 0.001*			
	T_6_	51.03 ± 5.69	60.74 ± 8.28	6.220	< 0.001*			
Anxiety and depression	T_1_	9.08 ± 3.59	13.73 ± 3.46	6.054	< 0.001*	^a^Group × Time	10.435	< 0.001*
	T_3_	7.21 ± 3.65	11.36 ± 3.42	5.410	0.010*			
	T_6_	7.05 ± 1.61	9.50 ± 3.81	3.781	< 0.001*			
Quality of life	T_1_	28.97 ± 1.67	28.20 ± 1.91	− 1.374	0.187	^a^Group × Time	5.540	0.021*
	T_3_	38.43 ± 2.99	36.38 ± 2.30	− 3.515	0.001*			
	T_6_	42.96 ± 2.65	38.46 ± 3.12	− 8.333	< 0.001*			
Functional exercise compliance	T_1_	66.40 ± 4.49	56.72 ± 3.31	NA	NA	^a^Group × Time	2.751	0.073
	T_3_	53.60 ± 3.85	45.27 ± 3.70					
	T_6_	45.98 ± 4.01	38.69 ± 3.25			^b^Group	209.855	< 0.001*

T_1_ (one month), T_3_ (three months), T_6_ (six months)

*NA* not applicable

^*^Statistically significant with *P* value < 0.05

^a^Group × Time, interaction effect between intervention and time

^b^Group, main effect for intervention

For self-efficacy and secondary outcomes, the interaction effects between intervention and time were significant, which means the effects of intervention varied depending on time. Therefore, it is of little significance to pay attention to the test results of main effects. Instead, we analyzed the simple effects of the intervention by performing independent sample t tests of two groups at three tracking points, respectively [20]. The results showed that intervention group’s self-efficacy and most secondary outcomes were significantly better than those of the control groups on 1, 3, and 6 months after surgery (p ≤ 0.01), while for quality of life at one month after surgery, the difference was not significant (*p* = 0.187 (see Table 3)).

For functional exercise compliance, the interaction terms between intervention and time were not significant. So we could focus on the main effect for intervention (p < 0.001), indicating that the difference between the two groups caused by the intervention was significant (Table 3).

## Discussion

A nurse-led, six-month SEEI was conducted to improve the effectiveness of participants’ rehabilitation following THR. This randomized controlled trial showed significant advantages of the SEEI over routine nursing for rehabilitation outcomes, including self-efficacy, joint function, psychological status, and long-term quality of life.

Self-efficacy is the target of the intervention. Strategies used in education and follow-ups were designed to enhance self-efficacy from its four main sources, that is, individual past experience, vicarious experience, verbal persuasion, and psychological monitoring. Previous interventions based on Bandura’s self-efficacy theory also achieved a result in enhancing confidence in self-management patients with chronic disease [26, 27]. Tzu-Ting Huang et al. conducted an empowerment education program on old adults with THR and significantly improved their self-care competence and self-efficacy[28]. Although based on different theories, there are similarities in the intervention strategies used in the two studies, for example, both of them applied the skills of guiding patients to set achievable goals and identifying past successful experience. So the education strategies are recommended in clinical nursing practice.

Few studies examined the variation tendency of exercise self-efficacy. A RCT, describing the growth trajectories of exercise self-efficacy in the elderly, pointed out participants would recalibrate their efficacy upon exposure to the actual exercise experience [29]. The six months of actual exercise supplemented performance-mastery experience, the most robust source of self-efficacy, and resulted in an apparent rise in both intervention and control groups [30] while the SEEI program, through face-to-face or telephone-based interventions, consolidated and facilitated self-efficacy furtherly, through education, problem-solving, and encouragement. In addition, the influence of self-efficacy may differ depending upon which stage of the exercise process the individual is currently in, and it is thought to be most vital in the initial stage of behavior [29]. Given the poor self-efficacy of patients who have recently undergone a hip replacement, it is necessary to provide such interventions from the early postoperative stage to maximize the positive effect of self-efficacy.

Patients’ compliance is a crucial predictor of the home-based rehabilitation effect [31]. The intervention group’s advantage in compliance reflected the effectiveness of SEEI on behavior change. The enhancement of self-efficacy could explain the better exercise compliance since it refers to individual confidence to complete the given task; greater self-efficacy allows patients to overcome challenges more easily. And among psychological barriers to compliance encompassing anxiety, depression, and hopelessness, self-efficacy is most likely to be intervened by medical staff [16]. Therefore, we recommend the self-efficacy-enhancing intervention program to be combined with routine care.

Furthermore, feedback and monitoring interventions also played a role. In the follow-ups, participants were asked to report the total numbers of home-based exercises they had performed, and positive feedback was given to the adherent behavior. The method of feedback and monitoring differed across studies, with daily activity logs, physiotherapist-supervised exercise classes, and group-based training demonstrating positive results [27, 32]. In fact, for patients with chronic diseases, such as cancer, diabetes, and dementia, supervised exercise interventions yielded benefits superior to non-supervised exercise programs in various outcomes, including quality of life and compliance to exercise and other physical and psychosocial outcomes [33]. And compared with face-to-face supervision, flexible tele-rehabilitation is helpful to reduce transmission of the coronavirus during the COVID-19 pandemic as well as allowing more patients, especially those after orthopedic surgeries, to access therapy and reduce healthcare costs [34]. Meanwhile, during five intervention sections, nurses offered exercise advice; caregivers were mobilized to provide encouragement, which provided participants emotional and practical assistance. Social support is believed to facilitate compliance and health [35], especially in patients with hip replacement, the population primarily composed of the elderly [36].

In the early postoperative stage, joint function was poor due to surgical trauma and pain, but it gradually improved over time. Patients in intervention group recovered more quickly than control group and reached a “good” or even “excellent” condition after six months. According to the internal reinforcement mechanisms, people will be more likely to continue exercising after noticing improvements in symptoms with exercise [37]. In the SEEI program, participants were reinforced by the direct effect of systematic functional exercise, including reducing pain, improving range of motion, and daily activities. Nurses’ endorsement also helped patients establish attributions between exercise and symptom improvements, contributing to better compliance and a virtuous circle.

Anxiety and depression was relatively common in patients three days after hip replacement [38]. Negative emotions, especially persistent anxiety, are potential risk factors for lasting postoperative pain and rehabilitation [39]. Moreover, persistent pain also caused emotional symptoms. Therefore, the early postoperative period deserves special attention, in which the overall physiological and psychological status was poor. The SEEI program is recommended for the superiority in decreasing anxiety and depression, relieving pain and improving joint function.

In this study, we also paid attention to patients’ postoperative activity and participation, a multidimensional concept containing the physiological, mental, and social aspects necessary for returning to daily life, as well as a critical indicator for successful rehabilitation [40]. According to Witjes and Yakushiji, patients are eager for activity and social participation following THR, which means that they yearn for the advanced activities of daily living, which are more sophisticated beyond those necessary to live independently, rather than basic expectations such as walking ability and pain relief [41, 42]. However, the pain, fatigue, and medical restriction impeded their return to daily activities. Based on patients from developed countries, a review found a great majority returned to sport and work after THR within a timeframe of 28 and 17 weeks, respectively [43]. In this study, participants still had a mild-to-moderate limitation on social participation six months past surgery, which may be related to the older age of subjects. Future research should focus on the implementation and effect test of the interventions to promote returning to daily life and work.

Joint function is the direct influential factor of quality of life for patients with hip replacement [44]. With the joint function getting better, their quality of life improved gradually. The intervention group had better long-term quality of life than control group, while the difference in the first month was not significant. As a measurement involving virous aspects of physiology and psychology, the improvements are often apparent three months to 1 year after surgery, rather than in the short term [45].

Main limitation of the study was the generalizability of the sample since participants were sampled from a 3a hospital in Guangdong Province, China (According to China's current *Hospital Grading Management Measures* and other provisions of the classification for medical institutions, 3a is the highest level in the classification system for hospitals in mainland China). Further studies of SEEI programs are needed in other regions and countries.

## Strengths and future study

The primary strength of the study was that we provided an operable and effective self-efficacy-enhancing intervention program for clinical nursing practice after hip replacement. Except for the first face-to-face education, the follow-ups were telephone-based, which was flexible and important to reduce transmission and healthcare costs, especially during the nowadays COVID-19 pandemic.

The present study has verified the effectiveness of the SEEI program on rehabilitation following THR. We also appeal for additional researches to apply this program and find empirical evidences related to different surgical approaches in order to act efficiently on rehabilitation outcomes and consequentially to improve a better joint function and reduce painful of patients in this condition.

## Conclusion

Patients with THR are recommended to do home-based exercise for 6 months. As multiple barriers exist, including hip pain, lack of knowledge, and specific guidance, they are short of confidence to overcome the challenges. Thus, a postoperative self-efficacy-enhancing intervention which provides knowledge, emotional support, and positive feedback has crucial influence. We recommend the SEEI to be routinely used in clinical practice and appeal for further efforts on promoting patients’ returning to daily work and life.

## Supplementary Information

**Additional file 1**. Characteristics and baseline status of participants.

## Data Availability

The datasets generated and analyzed during the study are available from the corresponding author by reasonable request.

## Abbreviations

THR: Total hip replacement
WHO-DAS II: World Health Organization Disability Assessment Schedule II
SEEI: Self-efficacy-enhancing intervention
