# Supplementary material for: Effectiveness of self-efficacy-enhancing interventions on rehabilitation following total hip replacement: a randomized controlled trial with six-month follow-up
Source: J Orthop Surg Res. 2022 Apr 10;17:225. doi: 10.1186/s13018-022-03116-2 (PMC8995056; doi:10.1186/s13018-022-03116-2)
Supplement: Supplementary file 1 — Additional file 1. Characteristics and baseline status of participants. [file 13018_2022_3116_MOESM1_ESM.docx]

**Supplemental Table S1. Characteristics and baseline status of participants (*n* = 150)**

| Variable | Intervention group (*n* =76)  *n* (%)/*X*±*S* | Control group (*n* =74)  *n* (%)/*X*±*S* | *χ^2^/t* | *P* |  |
| --- | --- | --- | --- | --- | --- |
| Work status |  |  | 9.753 | 0.244 |  |
| Unemployed | 28 (36.8) | 36 (48.6) |  |  |  |
| Retired | 23 (30.3) | 18 (24.3) |  |  |  |
| Employed | 25 (32.9) | 20 (27.1) |  |  |  |
| Per capita monthly income (yuan) |  |  | 8.528 | 0.126 |  |
| <1000 | 4 (5.3) | 11 (14.9) |  |  |  |
| 1000–1999 | 8 (10.5) | 14 (18.9) |  |  |  |
| 2000–2999 | 30 (39.5) | 19 (25.7) |  |  |  |
| 3000–3999 | 18 (23.7) | 15 (20.3) |  |  |  |
| >4000 | 16 (21.0) | 15 (20.3) |  |  |  |
| Health care payment type |  |  | 7.424 | 0.087 |  |
| Self-paying | 3 (3.9) | 5 (6.8) |  |  |  |
| Free medical care | 0 | 2 (2.7) |  |  |  |
| New rural cooperative medical scheme | 37 (48.7) | 46 (62.2) |  |  |  |
| Urban medical insurance | 30 (39.5) | 19 (25.7) |  |  |  |
| Commercial insurance | 6 (7.9) | 2 (2.6) |  |  |  |
| Operative site |  |  | 1.614 | 0.446 | |
| Left | 37 (48.7) | 31 (41.9) |  |  | |
| Right | 39 (51.3) | 43 (58.1) |  |  |  |
| Prosthetic material |  |  | 3.068 | 0.216 | |
| Titanium alloy | 0 | 2(2.7) |  |  | |
| Biological ceramics | 56 (73.7) | 58 (78.4) |  |  |  |
| Ultra-high molecular weight polyethylene | 20 (26.3) | 14 (18.9) |  |  |  |
| Preoperative complications |  |  | 1.915 | 0.384 | |
| None | 23 (30.3) | 23 (31.1) |  |  | |
| A single complication | 38 (50.0) | 30 (40.5) |  |  |  |
| Multiple complications | 15 (19.7) | 21 (28.4) |  |  |  |
| Self-efficacy of rehabilitation | 55.30 ± 6.09 | 55.20 ± 6.09 | -0.073 | 0.942 | |
| Hip function | 40.84 ± 8.29 | 42.96 ± 7.57 | 1.213 | 0.228 | |
| Activity and participation | 112.75 ± 11.17 | 109.18 ± 12.08 | -1.804 | 0.075 | |
| Anxiety and depression | 17.38 ± 3.93 | 18.25 ± 5.31 | -0.867 | 0.389 | |
| Quality of life | 24.27 ± 2.89 | 24.60 ± 2.40 | 0.565 | 0.573 | |
